# REVOLUTION (Routine EValuatiOn of people LivIng with caNcer)—Protocol for a prospective characterisation study of patients with incurable cancer

**DOI:** 10.1371/journal.pone.0261175

**Published:** 2021-12-16

**Authors:** Rebekah Patton, Jane Cook, Erna Haraldsdottir, Duncan Brown, Ross D. Dolan, Donald C. McMillan, Richard J. E. Skipworth, Marie Fallon, Barry J. A. Laird

**Affiliations:** 1 St Columba’s Hospice, Edinburgh, United Kingdom; 2 Academic Unit of Surgery, School of Medicine, University of Glasgow, Glasgow Royal Infirmary, Glasgow, United Kingdom; 3 Clinical Surgery, University of Edinburgh, Royal Infirmary of Edinburgh, Edinburgh, United Kingdom; 4 Institute of Genetics and Molecular Medicine, University of Edinburgh, Edinburgh, United Kingdom; Chang Gung Memorial Hospital at Linkou, TAIWAN

## Abstract

**Introduction:**

There is a pressing need for a holistic characterisation of people with incurable cancer. In this group, where quality of life and improvement of symptoms are therapeutic priorities, the physical and biochemical manifestations of cancer are often studied separately, giving an incomplete picture. In order to improve care, spur therapeutic innovation, provide meaningful endpoints for trials and set priorities for future research, work must be done to explore how the tumour influences the clinical phenotype. Characterisation of the host-tumour interaction may also provide information regarding prognosis, allowing appropriate planning of investigations, treatment and referral to palliative medicine services.

**Methods:**

**R**outine **EV**aluati**O**n of people **L**iv**I**ng with ca**N**cer (REVOLUTION) is a prospective observational study that aims to characterise people with incurable cancer around five key areas, namely body composition, physical activity, systemic inflammatory response, symptoms, and quality of life by developing a bio-repository. Participants will initially be recruited from a single centre in the UK and will have assessments of body composition (bio-impedance analysis [BIA] and computed tomography [CT]), assessment of physical activity using a physical activity monitor, measurement of simple markers of inflammation and plasma cytokine proteins and three symptom and quality of life questionnaires.

**Discussion:**

This study aims to create a comprehensive biochemical and clinical characterisation of people with incurable cancer. Data in this study can be used to give a better understanding of the ‘symptom phenotype’ and quality of life determinants, development of a profile of the systemic inflammatory response and a detailed characterisation of body composition.

## Introduction

There is an urgent need for characterisation of people with incurable cancer. In this group, where quality of life and symptom control are priorities, the physical and biochemical manifestations of cancer are often studied separately, giving an incomplete picture. In order to improve care, spur therapeutic innovation, provide meaningful endpoints for trials and direct future research, work must be done to explore how the tumour influences the clinical phenotype. Characterisation of this interaction may also provide prognostic information, allowing planning of investigations, treatment and referral to palliative medicine services [1].

There are five key areas that underpin a robust characterisation–body composition, physical function, the systemic inflammatory response, symptom phenotype and quality of life. The relationships between these areas are complex and not fully understood [2, 3].

Body composition is a priority, given the high prevalence of cancer cachexia- a syndrome encompassing metabolic and nutritional changes, weight loss and functional impairment [4]. Cachexia is common in people with gastrointestinal, lung and colorectal cancers however its true prevalence is unknown [5]. Despite a proliferation of literature regarding cachexia, fundamental questions remain unanswered. For example–does cachexia present similarly across tumour types? Kays et.al. found that even within a homogenous group of patients with pancreatic cancer three distinct cachexia phenotypes could be identified [6]. The links between body composition and physical function are also unclear. Recent trials of Anamorelin, a Ghrelin receptor agonist, in people with cachexia and lung cancer showed that although lean mass improved compared to placebo, hand grip strength did not, suggesting a non-linear relationship between lean mass and physical function [7, 8].

It has previously been shown that reductions in lean mass (muscle), body weight (muscle, fat, water and connective tissue) and degree of BMI have been associated with survival (prognosis) in patients with cancer [9–11]. The prospective study described below aims to further elucidate the relationship between survival and assessments of body composition and how these parameters relate to other clinical and biological features of incurable cancer.

Assessment of physical function is poorly documented in people with cancer [12]. Performance status (PS) (measured using the World Health Organisation Eastern Cooperative Oncology Group scale or Karnofsky Performance Status) is the most common measurement of physical function and is used to determine fitness for anti-cancer treatment. Despite this, PS is a highly subjective measure. Estimation of PS by oncologists differs significantly from patient assessment and is often more optimistic [13]. Moves have been made to improve objective assessment of physical function using wearable activity monitoring systems [14]. These systems have largely been used in trials involving cancer survivors, with limited use in incurable cancer [15].

The survival and spread of tumour cells has well established roots in the systemic inflammatory response (SIR) [16]. Bio-markers of the SIR have been shown to have association with survival in incurable cancer [17]. The SIR has also been shown to be associated with patient reported outcome measures and quality of life [18–21]. Work in this area has sought to characterise this relationship further through linking individual cytokines and symptoms [22–24].

Considering these areas, there remains sound argument for a characterisation of people with incurable cancer. The study described herein aims to meet this need. Characterisation is not a new concept, however this study targets an understudied population with repeated assessments, allowing for an understanding of the variation of clinical and biochemical factors over time. Given the need for characterisation of cachexia, initial data will be used to examine this syndrome.

## Methods

### Design

The REVOLUTION (**R**outine **EV**aluati**O**n of people **L**iv**I**ng with cancer) study is a prospective observational cohort study. Data and bio-samples collected as part of this study will be stored as part of the establishment of a novel biorepository. Full ethical approval has been given for the activities in this study (20/WS/0043) and the study will be conducted according to the principles of Good Clinical Practice and the Declaration of Helsinki.

### Population

Eligible participants will be recruited initially from a single centre palliative medicine service in the UK, with an aim for latter planned expansion to medical/surgical oncology services within the UK. Eligible participants will meet the following criteria-

- ≥18 years of age
- Diagnosis of incurable cancer (defined as clinical, histological, or radiological evidence of metastatic cancer, or receiving anti-cancer therapy with palliative intent).
- Ability to give informed consent

Participants will be excluded if they have a concomitant medical or psychiatric problem which, in the opinion of the investigator, would increase the risk of complication for the patient and/or investigator.

Patients with metastatic cancers which are potentially curable (i.e germ cell tumours, lymphoma) will be excluded. Anti-cancer therapy with palliative intent includes but is not limited to chemotherapy, radiotherapy, surgery, immunotherapy, hormone therapy and molecular target therapy.

## Assessments

Participants will then be invited to a baseline assessment, with follow up assessments at six weeks and twelve weeks post baseline. Participants will be free to withdraw at any time, without giving a reason.

At baseline, clinical data will be collected for each participant from the medical records. This will include demographic details including age and gender, and information regarding diagnosis date, tumour site, site of metastases and current and previous medications and anti-cancer treatment.

### Body composition

Routine oncology CT scans will be used as one of the methods of measuring body composition in the REVOLUTION study. CT scans are widely considered to be the gold standard for measurement of body composition, due to the fact that they can accurately and precisely differentiate between tissues of different densities [25, 26]. CT scans in this study will be evaluated at level of the L3 vertebrae (or at T10 for patients where chest CT only is available). The tissues will be identified, demarcated and quantified on each image using commercially available software. Serial CT images, obtained in routine oncology practice will be used to visualise the trajectory of lean mass.

Bio-impedance analysis offers a low-cost, radiation free alternative to CT and will be used at all three time points in the REVOLUTION study. BIA estimates total body water by measuring resistance to a small electrical current passed through the body [27]. This measurement can then be used to estimate lean mass and, by subtraction, body fat [28].

This study will incorporate measurements of body composition using both of the above methods in order to compare their suitability for use and practical application in people with incurable cancer. Height, weight, and Body Mass Index (BMI) measures will also be obtained. Bio-impedance measurements will be collected on all participants at all three study time points. Where available CT images obtained in routine oncology practice within 4 weeks of each time point will be analysed.

### Physical function

Each participant will be assessed by a health professional using the Karnofsky Performance Status Scale (KPS) [29]. The KPS provides an assessment of functional status using a scale with eleven points, each of which corresponds to a percentage score. 100% is normal no complaints; no evidence of disease, 0% is dead (see S1 Appendix) The KPS is used extensively in oncology to determine fitness for treatment and has been shown to have prognostic value in people with advanced cancer [30].

Participants will have an opportunity to subjectively describe their own physical function by completing the European Organisation for the Research and Treatment of Cancer–Quality of Life Questionnaire–C30 (EORTC QLQ-C30) [31]. The EORTC QLQ-C30 is a questionnaire with 30 items which measure five functional scales–(physical, role, emotional, cognitive and social) and three symptom scales–(pain, fatigue, nausea and vomiting).

An objective measurement of time spent undertaking different physical activities will be made by asking each participant to wear a small portable electronic activity monitor (Fitbit flex 2) for eight days. The flex 2 is a lightweight activity monitor worn in an unobtrusive rubber bracelet. Step count data from the Fitbit flex has been shown to correlate with clinician assessed performance status and may be useful in predicting re-admission to hospital following cancer surgery [32, 33]. This study will examine step count data and ‘minutes active data.’ The eight day wearing period accounts for six full days of 24hr wear with redundant days on either side where the patient is fitted with/ or returns the Fitbit. Previous studies have suggested that periods of no less than 6 days should be used for activity monitoring [34].

### Symptoms and quality of life

As mentioned above the EORTC-QLQ-C30 will be used in this study to record symptomatic information. To capture information regarding symptoms related to cachexia including social, emotional and physical aspects, the Functional Assessment of Anorexia/ Cachexia Therapy Scale (FAACT) [35] will be included. To capture information regarding nutrition, weight change and physical function the Patient Generated Subjective Global Assessment–Short Form (PGSGA-SF) will also be included [36]. Symptoms of depression and symptoms of dysphagia will be examined by specific questionnaires, namely, the Hospital Anxiety and Depression Scale (HADS) and the Eating Assessment Tool– 10 (EAT-10) [37, 38].

### Systemic inflammatory response

The known progressive derangement of normal inflammatory pathways in the body indicates that the biochemical factors that influence symptom generation and disease state in cancer fluctuate and change over time [39]. Therefore, participants in this study will have blood drawn at each of the three study time points. Blood samples will be analysed for known surrogate markers of the systemic inflammatory response including total white cell count and differential, C-reactive protein, lactate dehydrogenase and albumin. Samples will also be analysed for concentrations of multiple pro- and anti-inflammatory cytokines using Multiplex ELISA. (e.g., Interleukin (IL)-1α, IL-1β, IL-4, IL-6, IL-10, IL-17, Tissue Necrosis Factor-α). Samples of plasma and buffy coat will be stored with permission from the study participants for future use as part of a newly established research bio-repository. Where possible, research blood samples will be collected at the same time as routine samples. The choice of interleukins and tumour necrosis factors being examined in the current study has been informed by our recent work in appraising the literature as regards the relationship between circulating cytokines and symptoms and quality of life in patients with incurable cancer [40].

## Procedure

Participants will be identified by health professionals involved in their direct care and approached for consent by the study team prior to specific screening being conducted. They will be issued with a copy of the study information sheet and given as much time as required to decide to participate in the study. Participants will provide written informed consent to participate in this study and for their data and bio-samples to be stored for use in further studies in a newly established research bio-repository.

### Data management

Data will be collected on case record forms (CRFs) and transferred to a secure online database where participants are identified with a unique trial number. Ten percent data checks will be carried out for entry of data from paper CRFs into the electronic database. Electronic data will be routinely checked for accuracy. Standard operating procedures regarding recording and reporting of study data issued by the trial sponsor (ACCORD/NHS Lothian), Good Clinical Practice guidelines and data protection laws will be adhered to.

### Initial phase

**Fig 1:**
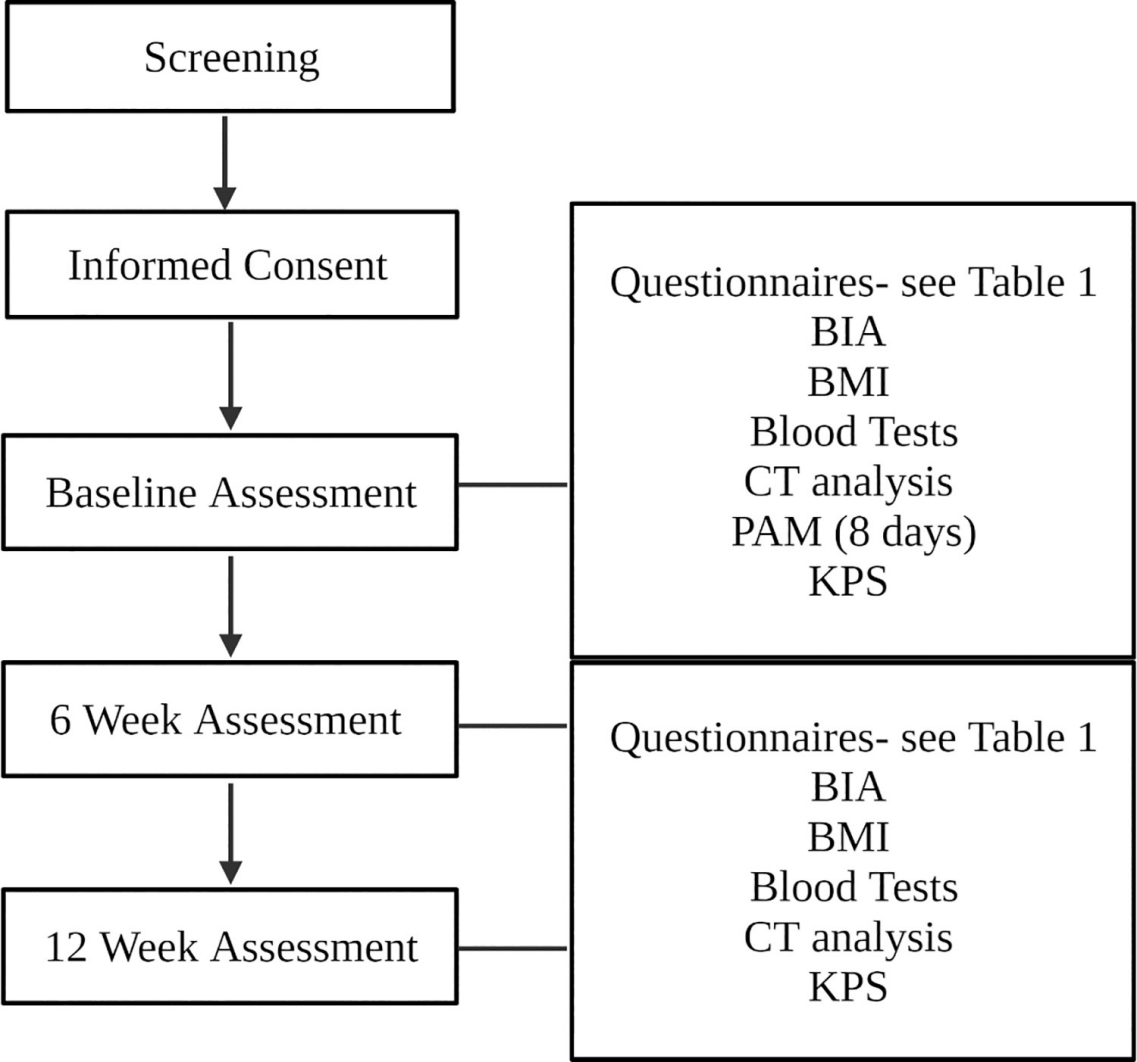
REVOLUTION study schematic.

The initial phase of this study will be conducted within a single centre setting. This phase will allow assessment of study set-up, feasibility of study structure and appropriateness of study measures. Please see below Table 1 for a detailed list of study assessments, and Fig 1 for REVOLUTION study schematic.

**Table 1:** Study assessments and time points.

	Baseline Assessment	6 week Assessment	12 week Assessment
**Demographics**	• Gender, place of care at baseline, current medications, primary tumour site, site of metastases, previous and current treatment	• Current treatment, current medications	• Current treatment, current medications
**Body Composition**	• Bio-impedance measurement	• BIA	• BIA
	• CT measurement (CT scans obtained as part of routine oncology practice)	• CT if available	• CT if available
	• Height	• Height	• Height
	• Weight	• Weight	• Weight
	• BMI	• BMI	• BMI
**Physical Function**	• Karnofsky Performance Status	• KPS	• KPS
	• Physical Activity Monitor worn continuously for eight days		
**Systemic Inflammatory Response**	• Full Blood Count	• FBC	• FBC
	• Urea and Electrolytes	• U and E	• U and E
	• Albumin	• Albumin	• Albumin
	• Plasma for cytokines	• Plasma for cytokines	• Plasma for cytokines
	• Lactate dehydrogenase	• LDH	• LDH
**Quality of Life and Symptoms**	• EORTC-QLQ-C30	• EORTC-QLQ-C30	• EORTC-QLQ-C30
	• FAACT	• FAACT	• FAACT
	• PGSGA-SF	• PGSGA-SF	• PGSGA-SF
	• HADS	• HADS	• HADS
	• EAT-10	• EAT-10	• EAT-10

## Discussion

People with incurable cancer are an under-studied population. The factors that determine the lived experience of incurable cancer are multiple, complex, and often studied in isolation. This study aims to create a comprehensive biochemical and clinical characterisation of this population, gathering data in five key areas–body composition, physical function, symptoms and quality of life, and the systemic inflammatory response.

Taking into account the five areas of study there are several key outcomes that the data in this study will contribute towards- including: A better understanding of the ‘symptom phenotype’ and quality of life determinants in people with incurable cancer, the development of a profile of the systemic inflammatory response and better understanding of the associations between the SIR and symptoms. The data in the study will also contribute towards a more detailed characterisation of body composition in people with incurable cancer and the prevalence of cancer cachexia. A better understanding of the clinical and biochemical features in patients with cachexia and those at risk of cachexia has the potential to inform the development of meaningful trial endpoints for therapeutics, and the development of effective multimodal treatment strategies.

Ultimately the data and bio specimens collected in this study will form a novel research bio-repository.

### Trial status

This description of the study is in keeping with the approved version of the study protocol (version 0.01, 28 January 2020). The study has been open to recruitment since the 15^th^ of July 2020. Initial recruitment is expected to last for 12 months. The data and bio samples from this study will form a research bio-repository.

## Conclusion

There is a need for a robust characterisation of people living with incurable cancer. This study aims to characterise this group in order to set priorities for future research and to inform high quality evidence- based care.

## Supporting information

S1 Appendix Karnofsky performance status. (DOCX)
