# Supplementary material for: REVOLUTION (Routine EValuatiOn of people LivIng with caNcer)—Protocol for a prospective characterisation study of patients with incurable cancer
Source: PLoS One. 2021 Dec 16;16(12):e0261175. doi: 10.1371/journal.pone.0261175 (PMC8675681; doi:10.1371/journal.pone.0261175)
Supplement: S1 Appendix — (DOCX) [file pone.0261175.s001.docx]

| Able to carry on normal activity and to work, no special care needed | 100 | Normal, no complaints, no evidence of disease. |
| --- | --- | --- |
|  | 90 | Able to carry on normal activity, minor signs or symptoms of disease. |
|  | 80 | Normal activity with effort, some signs or symptoms of disease. |
| Unable to work; able to live at home and care for most personal needs; varying amount of assistance needed | 70 | Cares for self, unable to carry on normal activity or do active work. |
|  | 60 | Requires occasional assistance, but is able to care for most of his personal needs. |
|  | 50 | Requires considerable assistance and frequent medical care. |
| Unable to care for self; requires equivalent of institutional or hospital care; disease may be progressing rapidly. | 40 | Disabled; requires special care and assistance. |
|  | 30 | Severely disabled; hospitalisation is indicated although death not imminent. |
|  | 20 | Very sick; hospital admission necessary; active supportive treatment necessary. |
|  | 10 | Moribund; fatal processes progressing rapidly. |
|  | 0 | Dead |

**S1 Appendix – Karnofsky Performance Status**
